# Melatonin regulates microglial polarization to M2 cell via RhoA/ROCK signaling pathway in epilepsy

**DOI:** 10.1002/iid3.900

**Published:** 2023-06-14

**Authors:** Pingping Li, Xuefei Ji, Ming Shan, Yi Wang, Xingliang Dai, Mengyuan Yin, Yunlong Liu, Liao Guan, Lei Ye, Hongwei Cheng

**Affiliations:** ^1^ Department of Neurosurgery The First Affiliated Hospital of Anhui Medical University Hefei China; ^2^ First Clinical Medical College Anhui Medical University Hefei China

**Keywords:** epilepsy, melatonin, microglia, polarization, RhoA/ROCK

## Abstract

**Background:**

Melatonin (MEL), an endogenous hormone, has been widely investigated in neurological diseases. Microglia (MG), a resident immunocyte localizing in central nervous system is reported to play important functions in the animal model of temporal lobe epilepsy (TLE). Some evidence showed that MEL influenced activation of MG, but the detailed model of action that MEL plays in remains uncertain.

**Methods:**

In this study, we established a model of TLE in mice by stereotactic injection of kainic acid (KA). We treated the mice with MEL. Lipopolysaccharide, ROCK2‐knockdown (ROCK‐KD) and ‐overexpression (ROCK‐OE) of lentivirus‐treated cells were used in cell experiments to simulate an in vitro inflammatory model.

**Results:**

The results of electrophysiological tests showed that MEL reduced frequency and severity of seizure. The results of behavioral tests indicated MEL improved cognition, learning, and memory ability. Histological evidences demonstrated a significant reduction of neuronal death in the hippocampus. In vivo study showed that MEL changed the polarization status of MG from a proinflammatory M1 phenotype to an anti‐inflammatory M2 phenotype by inversely regulating the RhoA/ROCK signaling pathway. In cytological study, we found that MEL had a significant protective effect in LPS‐treated BV‐2 cells and ROCK‐KD cells, while the protective effect of MEL was significantly attenuated in ROCK‐OE cells.

**Conclusion:**

MEL played an antiepileptic role in the KA‐induced TLE modeling mice both in behavioral and histological levels, and changed MG polarization status by regulating the RhoA/ROCK signaling pathway.

## Highlights

• Melatonin (MEL) reduces frequency and severity of seizure.
• MEL regulates the ROCK2 signaling pathway rather than ROCK1 to promote microglial polarization toward an anti‐inflammatory direction.
• RhoA/ROCK2 signaling pathway may be important in the temporal lobe epilepsy modeling mice.

## Graphical abstract

1. Melatonin (MEL) reduces frequency and severity of seizure. 2. MEL regulates the ROCK2 signaling pathway rather than ROCK1 to promote microglial polarization toward an anti‐inflammatory direction. 3. RhoA/ROCK2 signaling pathway might be a potential mechanism in the status epilepticus modeling mice.

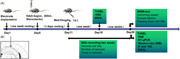

## 1 INTRODUCTION

Epilepsy is one of the most common neurological disorders, characterizing with abnormal synchronous discharges of the brain and thus causing recurrently spontaneous seizures. Epidemiological studies reported that more than 70 million individuals suffer from epilepsy worldwide.^1^, ^2^ With the development of techniques, vagus nerve stimulation (VNS) and deep brain stimulation (DBS) have been applied for the treatment of refractory epilepsy. However, such techniques are proved to partially control seizure symptoms, rather than to prevent the progression of neuropathies.^3^ An evidence suggests that seizure occurred in a circadian rhythm manner.^4^ Furthermore, a new technology, responsive neurostimulator system (RNS) introduces a novel view in epilepsy that is important to investigate the epileptic circadian rhythms to have predictions and corresponding treatment methods.^5^

Melatonin (MEL), an endogenous hormone, is predominantly secreted from pineal gland and retina^6^, ^7^, ^8^ that can bypass the blood‐brain barrier. It has been demonstrated that MEL played important roles in neurofunctional diseases by activating MEL receptors (MT1/2).^9^ Among the reports, MEL was widely reported in the pathogenesis and progression of epilepsy, indicating a protective role for the disease.^10^, ^11^ Besides, a clinical study found that the abnormal secretion of MEL resulted in circadian rhythm disorders and immune system dysfunctions.^12^ Meanwhile, a mechanism study showed that MEL changed the neurotransmission functions of glutamate and aminobutyric acid to regulate the electrical activity of neurons and to reduce oxidative stress responses in the alginate‐induced epilepsy of zebrafish.^13^ However, the specific antiepileptic mechanism of MEL is not fully elucidated. Therefore the MEL‐based treatment for patients with epilepsy is still a proof‐of‐concept.

The activation of immune response has been reported to be triggered in seizure.^14^ Microglia (MG) is a kind of immunocyte, predominantly localizing in central nervous system (CNS) and executing its functions via polarizations toward M1 and M2 phenotypes, which play proinflammatory and anti‐inflammatory effects, respectively.^15^ MG polarization has been extensively reported in a variety of CNS disorders, including stroke,^16^ traumatic brain injury,^17^ epilepsy,^18^, ^19^ and spinal cord injury.^20^ Furthermore, MG has also been proved to be important in temporal lobe epilepsy (TLE), mediating the secretion of CXCL8 through the NRLP3 inflammasome.^21^ Interestingly, the activity of MG is also functioned in a circadian manner.^22^ Therefore, we hypothesize that MEL may have a relationship with the activity of MG. Socodato et al. found that interfering with Rho GTPa signaling in MG directly resulted in neurodegeneration, suggesting that the activation of Rho‐related signaling pathways played an important role in neurodegenerative diseases.^23^ ROCK is a major downstream target of Rho, and two isoforms of ROCK (ROCK1/2) can phosphorylate specific substrates, such as myosin light chain kinase (MLCK) and myosin phosphatase subunit‐1 (MYPT1).^24^ The RhoA/ROCK signaling pathway has attracted increasing attentions for the involvement in numerous physiological processes, such as cytoskeletal rearrangement, inflammation, and cell migration.^25^ However, it is still lack of evidence whether MEL alleviates epilepsy and mediates MG polarization through RhoA/ROCK signaling pathway.

In this study, we hypothesize that MEL regulates the polarization of MG through RhoA/ROCK signaling pathway. We validated this hypothesis in an animal model and cell experiments. We think this MEL‐mediating signaling pathway assists our understanding about the functions of neuroinflammation in pathogenesis of epilepsy, and also provides a potential therapeutic target for preventing the neuropathy of epilepsy.

## 2 MATERIALS AND METHODS

### 2.1 Animal

Wide‐type male C57BL/6 mice, 6–8 weeks, were obtained from the Animal Experiment Center of Anhui Medical University. All mice were housed in a temperature‐ and humidity‐controlled condition with 12/12‐h light/dark cycles, with water and food provided regularly. All animal handling and experimental protocols were reviewed and approved by the Institutional Animal Care and Use Committee and Ethics Committee of First Affiliated Hospital of Anhui Medical University (approval No: LLSC20200107). The experimental procedures followed the United States National Institutes of Health Guide for the Care and Use of Laboratory Animals (NIH Publication No. 85‐23, revised 1996).

### 2.2 Kainic acid (KA)‐induced TLE modeling mice and MEL treatment

All mice were divided into four groups: control (CON), MEL, KA, and KA + MEL. An injection of KA (600 mL, 0.5 μg/μL; K0250; Sigma‐Aldrich) was performed stereotaxically (RWD Life Science) with a syringe at the following coordinates: anterior–posterior (AP) −0.12 mm, medial–lateral (ML) +0.14 mm, dorsal–ventral (DV) −0.18 mm,^26^ with the reference point of anterior fontanelle, which were based on the stereotaxic atlas of mice brain (the 2nd edition).

Diazepam (10 mg/kg) was used when the seizure sustained for 30 min.^27^ The mice with status epilepticus (SE) duration <30 min or Racine score < 4 were considered to be defective for modeling. Saline was used as a substitute in CON group. In the KA + MEL group, the MEL (M5250; Sigma‐Aldrich) was diluted in DMSO (dimethylsulfoxide, 10 mg/kg, pH 7.35) and injected into peritoneal cavity of mice on Day 3 after modeling.^28^, ^29^

### 2.3 Surgical procedures for electrophysiology test

Electrodes were implanted in the brain and muscle to record electroencephalograph (EEG) and electromyogram (EMG), respectively. The methods were described according to an established study.^30^ Briefly, after mice were anesthetized with pentobarbital sodium (50 mg/kg), two stainless steel screws with a diameter of 1 mm were inserted as cortical electrodes at the following coordinates: AP −1.3 mm, ML −2.0 mm, DV −1.6 mm. On the other hand, two insulated silver wires were placed on the quadratus muscle as electromyography electrodes. The other end of electrode was attached to a mini‐connector.

### 2.4 Intracranial video‐EEG recording

We recorded seizure grades using Racine scoring criteria (Stage 1, immobilization; Stage 2, facial or manual automatic hunchback; Stage 3, facial or manual automatic feeding; Stage 4, forelimb clonus and fall; Stage 5, generalized clonic convulsions with loss of right‐turn reflex) during the consecutive 15 days after modeling. EEG was used 0.1–70 Hz bandpass filtered and digitized at 400 Hz sampling rate using a digital system by subcutaneous head electrode embedding method (Pinnacle; Pinnacle Technology). Video EEG was continuously recorded daily (9:00–21:00) and seizure activity was analyzed offline by a trained specialist. According to EEG regulations, seizures were defined as an observation of regular spike clusters with seizure time >10 s, peak frequency >3 Hz, and amplitude > 3 times higher than the baseline.^31^ The acquisition and digitization of signals were via the Signep3‐OBI analysis software.

### 2.5 Behavioral monitor

MWM (Morris water maze) was used to test the behavioral differences in TLE modeling mice, including cognition, learning, and spatial memory. A circular pool with 150 cm width and 50 cm height was chosen. A platform with 10 cm diameter was placed in the target quadrant. In the place‐navigation trial, the mice were trained for 5 days (9:00–11:00). We put the mice in the pool from four different quadrants to find the target platform. In the spatial‐probe trial, after the platform was removed, we put the mice in pool from any of quadrant. We recorded path trace and calculated the number of times crossing platform as well as staying distance in the target quadrant within 120 s. Animal behavior was automatically tracked using a super maze behavior tracking software.

### 2.6 Histopathological evaluation

Paraffin‐embedded brain tissues were sectioned coronally (5 µm thick). Histopathological changes were analyzed by hematoxylin and eosin (H&E) staining (HampE, C0105S; Beyotime), Nissl staining (C0117; Beyotime), and TUNEL staining (C1086; Beyotime) according to the manufacturer's instructions. Pictures were taken using a fluorescence microscope.

### 2.7 Immumohistochemical staining

After deparaffinization of tissue sections, endogenous peroxidase blocker (PV‐9001; Zhongshan Golden Bridge) was added for 15 min to block endogenous peroxidase activity. After preincubation with 10% normal donkey serum (SL050; Solarbio) for 10 min in a microwave processor, slides were incubated overnight at 4°C with the primary antibodies, anti‐liver arginase antibody (Arg‐1, 1:200; Abcam) and anti‐inducible nitric oxide synthase antibody (iNOS, 1:200; Abcam). Then the slides were titrated with 100 µL enhanced enzyme‐labeled goat anti‐rabbit IgG polymer (PV‐9001; Zhongshan Golden Bridge) for 10 min. The results were detected with a light microscope.

### 2.8 Real‐time quantitative polymerase chain reaction (RT‐qPCR)

RNA was extracted using the Total RNA Isolation Reagent (BS258A; bio‐sharp) following the manufacturer's instructions, and RNA concentrations were measured with a nanodroplet spectrophotometer. First‐strand cDNA was synthesized from 500 ng RNA and used for qPCR by HiScript III RT SuperMix (Vazyme; R323‐01). RT‐qPCR was performed using SYBR green reagent (Q411‐02; SYBR Master Mix; Vazyme) on a Light Cycler 480 II machine. The gene expression was calculated according to the $2^{‐∆∆C_{q}}$ method. PCR primers for CD‐206, interleukin (IL‐10), found in inflammatory zone 1 (Fizz‐1), arginase 1 (Arg‐1), inducible nitric oxide synthase (iNOS), C‐C motif ligand 5 (CCL‐5), CCL‐3, tumor necrosis factor α (TNF‐α), and glyceraldehyde‐3‐phosphate dehydrogenase (GAPDH) were designed by referring to OriGene Technologies and literature^32^ and were summarized in Supporting Information: Table 1.

### 2.9 Cell culture

Mouse BV‐2 and HT22 cells were obtained from the Cell Bank of Chinese Academy of Sciences. The protocols for cell culture were described according to our previous study.^33^ Briefly, BV‐2 cells were pretreated with different concentrations of MEL for 2 h, and then were stimulated with lipopolysaccharide (LPS) (1 μg/mL) for 24 h. A conditioned medium (CM) containing targeted molecules was obtained by centrifugation with 500 g at 4°C. Then we transferred the CM to HT22 cell cultures and performed an incubation in 96‐well and 6‐well plates. Cell viability and apoptosis were detected after cell culture for 24 h. We found that MEL exceeding 50 μM produced cytotoxic effects in CCK‐8 trials, so the concentration of MEL at 50 μM was selected for the subsequent cytological experiments.

### 2.10 Cell viability assay (cells, ex vitro)

According to the manufacturer's protocol for the CCK‐8 kit (C0037; Beyotime), different concentrations of MEL were added to BV‐2 cells and the cytotoxicity was detected after incubation for 24 h. Then, 90 μL of fresh medium and 10 μL of CCK‐8 solution were added to each sample, respectively. Cells were incubated at 37°C for 60 min. Optical density values were measured with a microplate reader at 450 nm.

### 2.11 Establishment of ROCK2‐overexpression (ROCK‐OE) and ROCK2‐knockdown (ROCK‐KD) stable cells

BV‐2 cells were grown in six‐well plates (2 × 105 cells per well) and allowed to reach 40% confluence. The cells were then subjected to transfection with lentivirus following the manufacturer's protocol (OBIO Technology). Untransfected cells were filtered out by puromycin dihydrochloride (ST551; Beyotime).

ShRNA for ROCK2 and the negative control (NC) were cloned into the lenti‐KD vector pSlenti‐U6‐shRNA‐CMV‐EGFP‐F2A‐Puro‐WPRE to produce pSlenti‐U6‐shRNA (ROCK2)‐CMV‐EGFP‐F2A‐Puro‐WPRE. The ROCK2 was cloned into the OE vector PcSLenti‐CMV‐MCS‐3xFLAG‐PGK‐puro‐WPRE3 to produce PcSLenti‐CMV‐Rock2‐3xFLAG‐PGK‐puro‐WPRE3 (OBIO Technology). We verified ROCK2‐KD and ‐OE by Western blot (WB), and the core target sequences for shRNA and primers amplifying ROCK2 were listed in Table 2.^34^, ^35^

### 2.12 Immunofluorescence (IF) staining

Brain slides and BV‐2 cells on coverslips were blocked in 10% donkey serum for 1 h and 0.3% Triton X‐100 in PBS for 20 min at room temperature and probed with the primary antibodies overnight at 4°C,^36^ anti‐iNOS antibody (ab49999; Abcam), anti‐iba‐1 (ionized calcium binding adapter molecule 1) antibody (#17198; Cell Signaling Technology), anti‐liver Arginase antibody (ab239731; Abcam), respectively. After being washed, the slides were treated with Cy3‐labled secondary antibodies (GB21301/GB25303, 1:300/1:400; Sevicebio) for 2 h.

### 2.13 Flow cytometry (FCM)

BV‐2 cells across different groups (CON, DMSO, LPS, LPS + MEL, CON + LPS + MEL, ROCK‐KD + LPS + MEL, and ROCK‐OE + LPS + MEL) were subjected to FCM. After cell digestion and centrifugation at 500 g for 5 min to collect cells, flow external standard antibodies FITC‐CD86 (11‐0862‐81; Invitrogen), APC‐Cy7‐CD45 (A15395; Abcam), and efluor‐450‐CD11b (48‐0112‐82; Invitrogen) were added, treating for 1 h at 4°C. Then the cells were fixed with IC fixation buffer (00‐8222‐49; Invitrogen) and permeabilization buffer (00‐8333‐56; Invitrogen) for 25 min at room temperature. APC‐CD206 (17‐2061‐82; Invitrogen) was added to label cells after fixation of the broken membrane. Controls were performed according to the instructions. All antibodies were added using rat serum (ratio of rat serum and antibody: 3:1) for blockade and cells were sorted by CyTExpert 2.4 cell sorter.

### 2.14 WB

Total proteins from tissues and cells were extracted using a RIPA buffer kit (P0013B; Beyotime) according to the manufacturer's protocols. The procedures of WB were performed according to a previous study.^33^ Briefly, the membranes were blocked with 5% bovine serum albumin (BSA) which was dissolved in Tris‐buffered saline with Tween‐20 for 1 h at room temperature. Then the membranes were labeled with primary antibodies, anti‐myosin light chain 2 (MLCK2) antibody (1:1000, #3672; Cell Signaling Technology), anti‐phospho‐myosin light chain 2 (p‐MLCK2) antibody (1:1000, #3674; Cell Signaling Technology), anti‐MYPT1 antibody (1:1000, #2634; Cell Signaling Technology), anti‐p‐MYPT1 (Thr696) antibody (1:1000, PA5‐38297; Invitrogen), anti‐ROCK1 antibody (1:1000, AF0276; Beyotime), anti‐RhoA antibody (1:1000, AF2179; Beyotime), anti‐ROCK2 antibody (1:1000,#47012,Cell Signaling Technology), and GAPDH antibody (1:1000, AF1186; Beyotime), at 4°C overnight, respectively, followed by incubations with corresponding secondary IgG (H + L) (peroxidase/horse radish peroxidase conjugated) antibodies (E‐AB‐1001; Elabscience) at different dilutions for 2 h at room temperature. The protein bands were visualized and captured using a Tanon 1600R imaging system. The optical density of each band was quantified using ImageJ software and normalized to the intensity of GAPDH.

### 2.15 Statistical analysis

SPSS software (version 19.0) was used for statistical analysis. All enumeration data were given as the mean ± standard deviation (SD) and were analyzed by Student's *t* test or a Kruskal–Wallis test between two groups, and by one‐way analysis of variance analysis when group > 3. *p* < .05 was considered as the statistical significance.

## 3 RESULT

**Figure 1:**
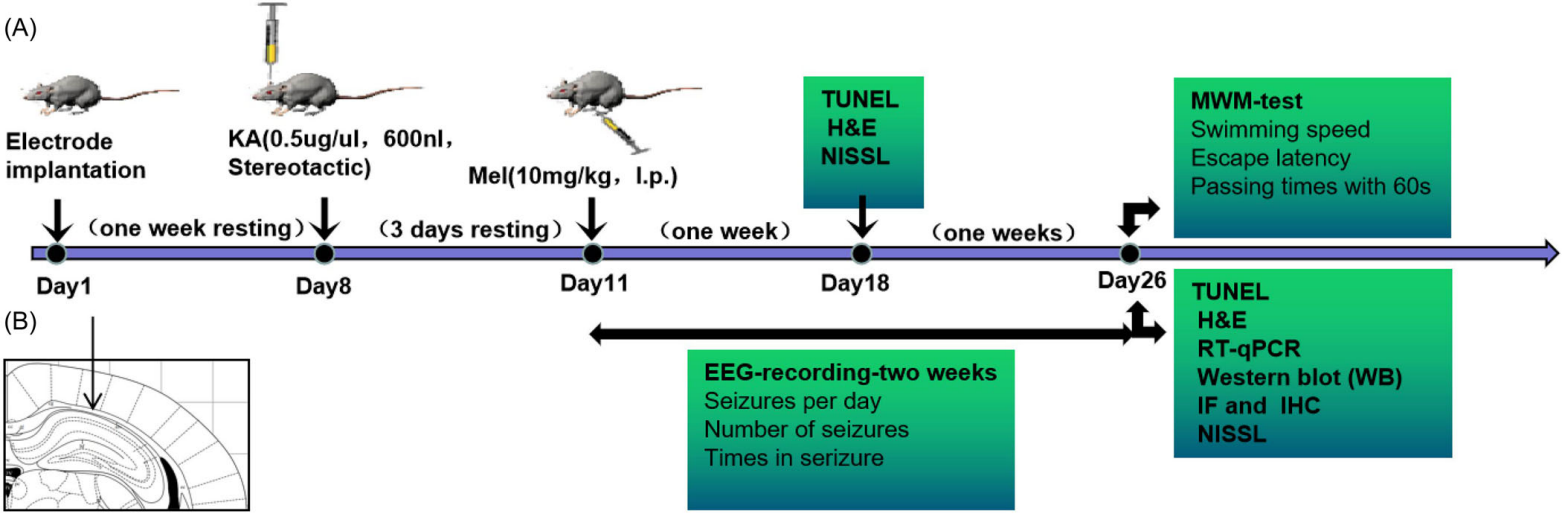
Experimental design. (A) Mice were operated and recovered for 7 days. Kainic acid (KA) was stereotaxically injected into the hippocampus. After 3 days of resting, melatonin (MEL) was injected intraperitoneally. Electroencephalograph (EEG) recordings were then recorded after treatment of MEL. While learning and cognitive abilities were evaluated by Morris water maze (MWM) test (15 days after MEL injection). In addition, brain tissue samples were harvested for biochemical testing or pathological analysis on Days 7 and 15 after MEL injection. (B) The scheme for stereotactic injection of the drug.

We established an epilepsy model in mice by stereotactic injection of KA, and treated the mice with MEL. The results in behavioral tests showed that MEL improved cognition, learning, memory abilities in TLE modeling mice. The cytological results showed that MEL reduced neuronal apoptosis in the hippocampus of epileptic mice by inhibiting microglial polarization to M1 proinflammatory direction and promoting microglial polarization to M2 direction. MEL alleviated seizures probably through reverse regulation of RhoA/ROCK2 signaling pathway. The experimental design is depicting at Figure 1.

### 3.1 MEL alleviated seizure and recovered behaviors in SE modeling mice

**Figure 2:**
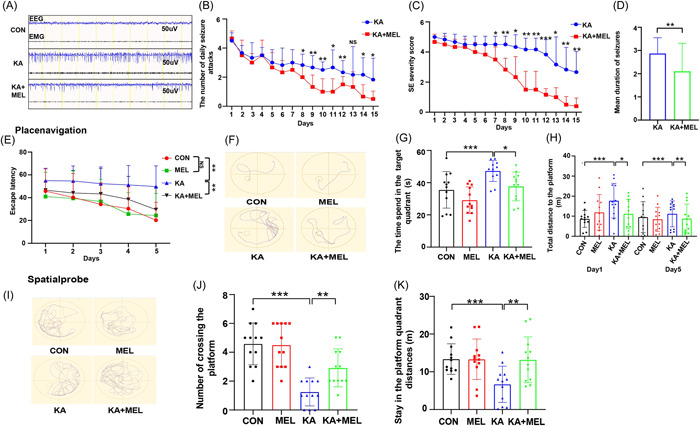
Behavior monitor of mice in different groups. (A) Electroencephalograph (EEG) and electromyogram (EMG) records within 40 s in CON, kainic acid (KA), and KA + melatonin (MEL) groups. (B) The number of daily seizure attacks within 15 days in KA and KA + MEL groups. (C) The severity score of epilepsy within 15 days in KA and KA + MEL groups. (D) The mean daily seizure frequency within 15 days in KA and KA + MEL groups (F_KA + MEL_ = 3.138, vs. KA). (E) Escape latency time in 5 days (F_KA_ = 18.93, vs. CON, F_KA + MEL_ = 9.152, vs. KA). (F) Water maze navigation experiment. (G) The time spend in the target quadrant (s) (F_KA_ = 2.236, vs. CON; F_KA + MEL_ = 1.846, vs. KA). (H) Distance to the platform on Day 1 (F_KA_ = 2.234, vs. CON; F_KA + MEL_ = 1.402, vs. KA) and Day 5 (F_KA_ = 1.341, vs. CON; F_KA + MEL_ = 1.002, vs. KA). (I) Water maze spatial exploration experiment. (J) Number of crossing the platform (F_KA_ = 2.236, vs. CON; F_KA + MEL_ = 1.846, vs. KA). (K) Stay in the platform quadrant total distances (F_KA_ = 1.410, vs. CON; F_KA + MEL_ = 1.598, vs. KA). (*p < .05; **p < .01; ***p < .001).

We used EEG and EMG for detecting the electrical activities in different groups. The results showed that the frequency of spike wave and the width of peak in the KA group significantly increased in comparison with those in the KA + MEL group, indicating that MEL significantly reduced the frequency and degree of seizure (Figure 2A). After an intraperitoneal injection of MEL for a consecutive 7 days, the numbers of seizure and the severity score in KA + MEL group decreased (Figure 2B,C). After 15 days, the mean duration of seizure in KA + MEL group decreased compared with that in KA group. Meanwhile, we found that the occurrence of seizure was greater at the daytime than at the nighttime. The most frequent time of seizure was in the midday hours, when MEL secretion was lowest in animals (Figure 2D). In place‐navigation trials of MWM, we found that the escape latency, the time spend in target quadrant and the distance to the platform decreased in the KA + MEL group in comparison with those in KA group (Figure 2E–H). In spatial‐probe test, the numbers of crossing target platform and staying distance in platform quadrant increased in the KA + MEL group in comparison with those in KA group (Figure 2I–K), These findings suggested that MEL improved the cognition and learning abilities in TLE modeling mice.

### 3.2 MEL‐protected hippocampal neuron

**Figure 3:**
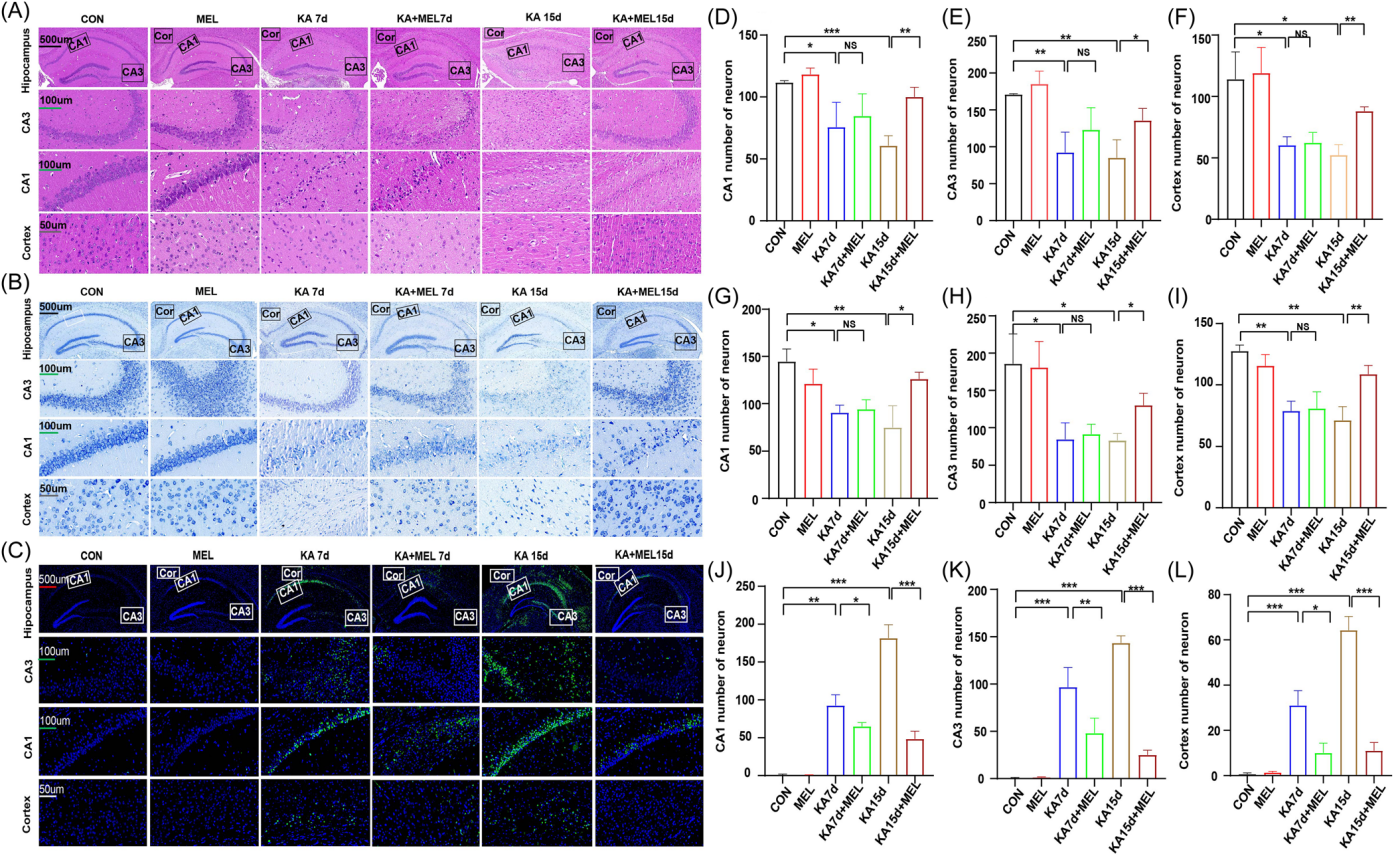
Histological tests on cerebral cortex and hippocampus in different groups. (A) Hematoxylin and eosin (H&E) staining on cortex and hippocampus (CA1, CA3) regions. (B) Nissl staining on cortex and hippocampus (CA1, CA3) regions. (C) TUNEL staining on cortex and hippocampus (CA1, CA3) regions. (D–F) Statistics of neuron numbers in CA1, CA3, and cortex regions on H&E staining; (D: F_KA7d_ = 171.6, vs. CON; F_KA15d_ = 28, vs. CON; F_KA15d+MEL_ = 1.701, vs. KA15d. E: F_KA7d_ = 758.3, vs. CON; F_KA15d_ = 603, vs. CON; F_KA15d + MEL_ = 2.281, vs. KA15d. F: F_KA7d_ = 10.77, vs. CON; F_KA_15d = 6.899, vs. CON; F_KA15d + MEL_ = 5.564, vs. KA15d). (G–I) Statistics of neuron numbers in CA1, CA3, and cortex regions on Nissl staining; (G: F_KA7d_ = 1.870, vs. CON; F_KA15d_ = 4.440, vs. CON; F_KA15d + MEL_ = 10.28, vs. KA15Day. H: F_KA7d_ = 2.179, vs. CON; F_KA15d_ = 12.41, vs. CON; F_KA15d+MEL_ = 3.029, vs. KA15Day. I: F_KA7d_ = 2.108, vs. CON; F_KA15d_ = 4.0, vs. CON; F_KA15d+MEL_ = 2.464, vs. KA15Day). (J–L) Statistics of neuron numbers in CA1, CA3, and cortex regions on TUNEL (J: F_KA7d_ = 201.3, vs. CON; F_KA15d_ = 308.3, vs. CON; F_KA7d+MEL_ = 8.053, vs. KA7Day; F_KA15d+MEL_ = 2.846, vs. KA15Day. K: F_KA7d_ = 1300, vs. CON; F_KA15d_ = 175, vs. CON; F_KA7d+MEL_ = 1.720, vs. KA7Day; F_KA15d+MEL_ = 2.333, vs. KA15Day. L: F_KA7d_ = 129, vs. CON; F_KA15d_ = 109, vs. CON; F_KA7d+MEL_ = 2.263, vs. KA7Day; F_KA15d+MEL_ = 2.795, vs. KA15Day.). (*p < .05; **p < .01, ***p < .001).

H&E, Nissl, and TUNEL staining of hippocampal tissue were performed to observe the hippocampal neuron damages after TLE modeling and MEL treatment. We found that the numbers of neuron in hippocampus (CA1, CA3) and Cortex region decreased at 7 days and 15 days after TLE modeling. After an intraperitoneal injection of MEL for 15 days, the numbers of neuron recovered (Figure 3A,D–F). The results of Nissl staining demonstrated a similar result (Figure 3B,G–I). In TUNEL staining, the numbers of neuron recovered after an intraperitoneal injection of MEL for 7 days (Figure 3C,J–L). Hippocampal neuronal injury was severe with the prolonged seizure durations. The numbers of neuron in the hippocampus (CA1, CA3) and cortex region significantly reduced in the KA group, while MEL treatment significantly increased the numbers of neurons in those areas.

### 3.3 MEL altered MG polarization and affected RhoA/ROCK signaling pathway

**Figure 4:**
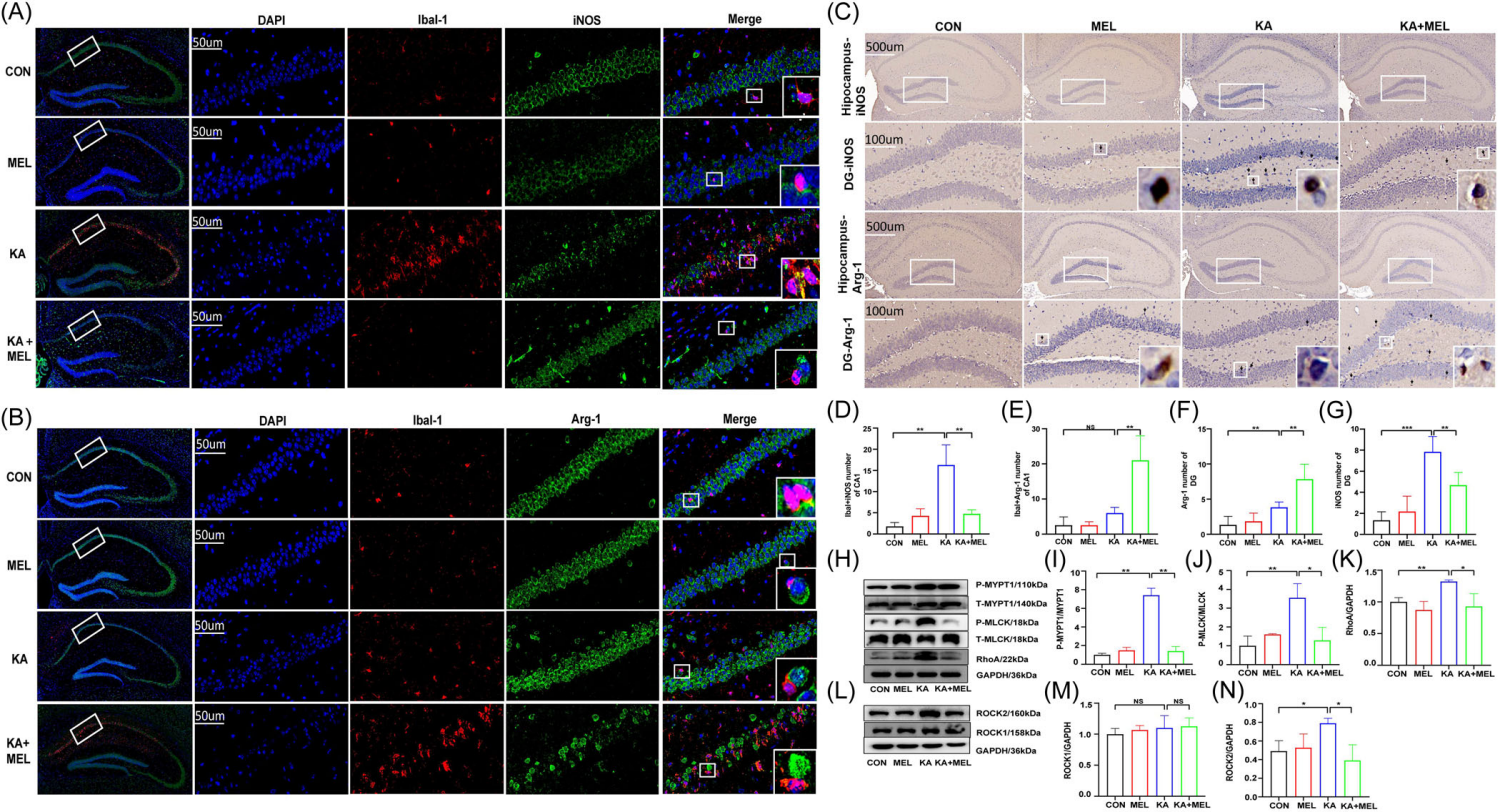
Immunofluorescent (IF) and immunohistochemical (IHC) staining on cerebral cortex and hippocampus in different groups. (A and B) IF staining for M1 and M2 cells in CA1 region of the hippocampus, respectively. (C) IHC staining for M1 and M2 cells in DG region of the hippocampus. Statistics of co‐staining of iba‐1 and iNOS (F_KA_ = 25, vs. CON; F_KA+MEL_ = 25, vs. KA) (D) as well as iba‐1 and Arg‐1 (F_KA+MEL_ = 18.25, vs. KA) (E) in CA1 and Statistics of Arg‐1 (F_KA_ = 2.588, vs. CON; F_KA+MEL_ = 8.059, vs. KA) (F) as well as iNOS (F_KA_ = 3.250, vs. CON; F_KA+MEL_ = 1.477, vs. KA) (G) in DG regions in different groups. Protein levels in RhoA/ROCK signaling pathway. (H) Western blot (WB) tests for RhoA, p‐MYPT1, t‐MYPT1, p‐MLCK, and t‐MLCK proteins. Statistics for p‐MYPT1/t‐MYPT1 ratio (I) (F_KA_ = 18.26, vs. CON; F_KA+MEL_ = 2.303, vs. KA), p‐MLCK/t‐MLCK ratio (J) (F_KA_ = 2.093, vs. CON; F_KA+MEL_ = 1.158, vs. KA) and RhoA/GAPDH ratio (K) (F_KA_ = 6.234, vs. CON; F_KA+MEL_ = 59.41, vs. KA) expressions in different groups; (L) WB tests for ROCK1 and ROCK2 proteins. Statistics for ROCK1/GAPDH ratio (M) and ROCK2/GAPDH ratio (N) (F_KA_ = 1.658, vs. CON; F_KA+MEL_ = 7.650, vs. KA) in different groups. (*p < .05; **p < .01, ***p < .001).

The results of immunostaining showed that the positive staining for M1 cells (double staining of iba‐1 and iNOS) (Figure 4A,D) significantly increased, while the positive staining for M2 cells (double staining of iba‐1 and Arg‐1) (Figure 4B,E) decreased at CA1 region in KA group, respectively. However, after the treatment with MEL, the content of immunostaining for M1 cells decreased, while that for M2 cells increased in comparison with those in the KA group. Meanwhile, we performed immunohistochemical staining for M1 and M2 cells at dentate gyrus (DG) region. The results were similar to that of immunostaining at CA1 region (Figure 4C,F,G), indicating that MEL had impacts on the MG polarization in TLE modeling and MEL treatment.

Meanwhile, RT‐qPCR was performed to detect the content of cytokines in the hippocampal tissues. The results showed that the markers concerning M1 (CCL‐5, iNOS, CCL‐3, TNF‐α) significantly increased in KA group, but decreased after MEL treatment (Supporting Information: Figure 1A–D). In contrast, the markers concerning M2 (Arg‐1, CD206, IL‐10, Fizz‐1) significantly increased after MEL treatment than those in the KA group (Supporting Information: Figure 1E–H). The results indicated that MEL inhibited the levels of proinflammatory cytokines and promoted the levels of anti‐inflammatory cytokines in the SE modeling mice.

At the protein level, the expressions of RhoA/ROCK, p‐MLCK and p‐MYPT1 were determined by WB in different groups (Figure 4H,l). We found that protein levels of p‐MLCK, p‐MYPT1, RhoA, and ROCK2 were downregulated after MEL treatment in comparison with those in the KA group (Figure 4I–K,N). However, the subunits of ROCK1 protein did not change after TLE modeling and MEL treatment (Figure 4M).

### 3.4 MEL reduced toxicity on BV2 and HT22 cells

CCK‐8 tests were used to detect the toxicity of MEL on HT22 cells and LPS‐stimulated BV‐2 cells. The results demonstrated that MEL had no effect on BV‐2 cells (Supporting Information: Figure 2A) and the toxicity of CM‐stimulated HT22 cells showed a reduced trend with the increases of MEL concentration, indicating that MEL reduced the neurotoxic effect on HT22 cells (Supporting Information: Figure 2B). Meanwhile, the results of FCM for Annexin V had a similar phenomenon with that in the CCK‐8 tests (Supporting Information: Figure 2C). The apoptotic level of HT22 cells decreased with the elevated concentrations of MEL (Supporting Information: Figure 2D).

### 3.5 MEL affected RhoA/ROCK signaling pathways in BV‐2 cells and changed the polarization in BV‐2 cells

**Figure 5:**
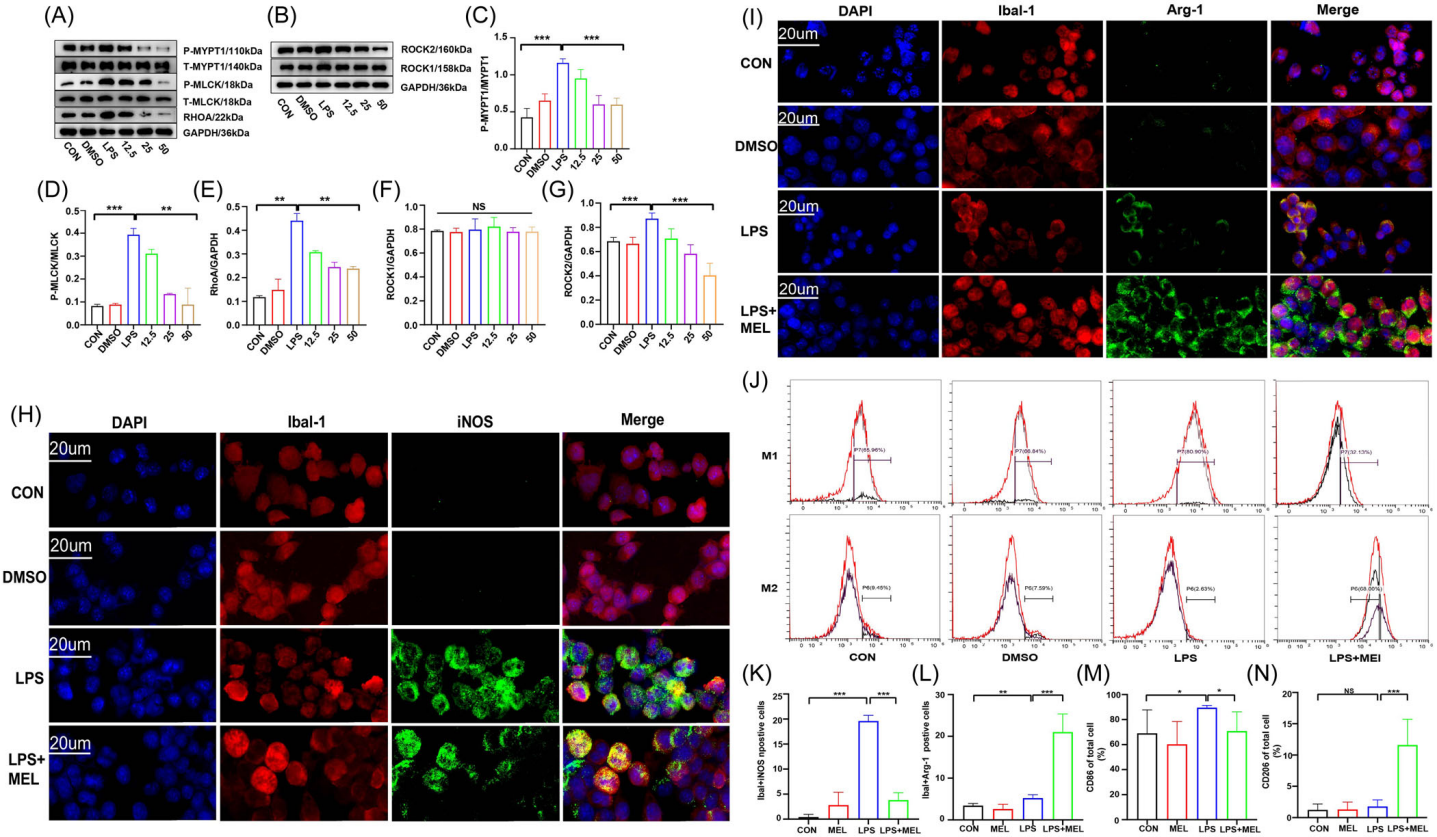
The expression of RhoA/ROCK signaling pathway in cells. The expression of p‐MYPT1, p‐MLCK, RhoA (A) and ROCK1/2 (B) proteins expression; statistics for p‐MYPT1/t‐MYPT1 ratio (C) (F_(2,6)_ = 49.03, F_(3,8)_ = 23, versus LPS), p‐MLCK/t‐MLCK ratio (D) (F_(2,3__)_ = 229.8, F_(3,4)_ = 26.40, vs. LPS) and RhoA/GAPDH ratio (E) (F_(2,3)_ = 63.44, F_(3,4)_ = 48.58, vs. LPS) in different groups; Statistics for ROCK1/GAPDH ratio (F) and ROCK2/GAPDH ratio (G) (F_(2,6)_ = 56.40, F_(3,8)_ = 54.99, vs. LPS) in different groups. (**p < .01; ***p < .001). Immunofluorescent (IF) staining and flow cytometric (FCM) analysis in cells. IF staining for M1‐related biomarker (iba‐1 and iNOS) (H, K: F_LPS_ = 2.333, vs. CON; F_LPS+MEL_ = 27.14, vs. LPS) and M2‐related biomarkers (iba‐1 and Arg‐1) (I, L: F_LPS_ = 4.333, vs. CON; F_LPS+MEL_ = 1.692, vs. LPS) in BV‐2 cells; Proportion of CD86 (M1 cell) and CD206 (M2 cell) in BV‐2 cells (J, M: F_LPS_ = 1.374, vs. CON; F_LPS+MEL_ = 9.425, vs. LPS; N: F_LPS+MEL_ = 91.54, vs. LPS).

We validated the proteins in RhoA/ROCK signaling pathway in BV‐2 cells by WB (Figure 5A,B). The results showed that protein levels of p‐MYPT1 (Figure 5C), p‐MLCK (Figure 5D), RhoA (Figure 5E), and ROCK2 (Figure 5G) significantly increased after LPS application, but decreased after MEL treatment. Besides, the subunits of ROCK1 protein did not change after the applications of LPS and MEL, respectively (Figure 5F).

In the IF staining for M1 and M2‐related biomarkers in BV‐2 cells (Figure 5H,I), we found that the co‐staining of iba‐1 and iNOS significantly increased after LPS application (Figure 5H,K), but decreased in the MEL + LPS group. Meanwhile, the co‐staining of iba‐1 and Arg‐1 had a diverse phenomenon (Figure 5I,L), indicating that MEL promoted the polarization of BV‐2 cell to M2 cells and meanwhile inhibited the M1 cell polarization. After BV‐2 cells were treated with MEL and LPS, the results showed that the proportion of CD86 significantly increased after the LPS application (Figure 5J,M), but decreased in MEL + LPS group. Meanwhile, the proportion of CD206 increased in the MEL + LPS group (Figure 5J,N).

We simultaneously detected the messenger RNA (mRNA) levels of inflammatory cytokines in BV‐2 cells. The results were consistent with those in animal experiments. M1 cell‐related proinflammatory cytokines (TNF‐α, CCL‐3, CCL‐5, iNOS,) significantly downregulated in LPS + MEL group than those in the LPS group (Supporting Information: Figure 3A–D). In contrast, M2 cell‐related anti‐inflammatory cytokines (Fizz‐1, IL‐10, CD206, Arg‐1) significantly upregulated in LPS + MEL group than those in the LPS group (Supporting Information: Figure 3E–H).

### 3.6 Mel changed the polarization direction of ROCK‐KD and ROCK‐OE inflammatory BV‐2 cells via RhoA/ROCK signaling pathways

**Figure 6:**
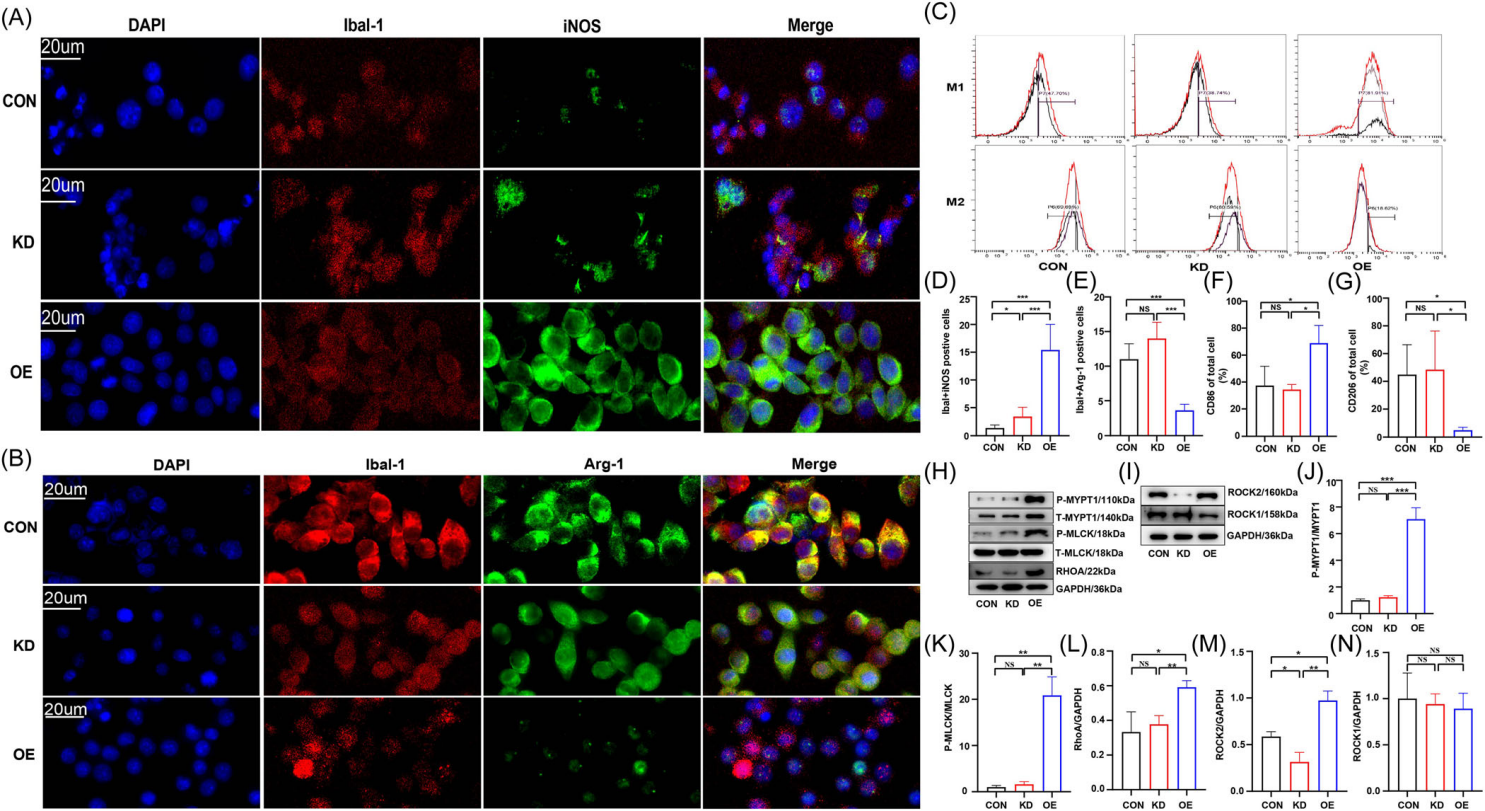
Expression of M1 and M2 biomarkers in ROCK2‐knockdown (ROCK‐KD) and ROCK2‐overexpression (ROCK‐OE) BV2 cell. (A, D: F_KD_ = 9.333, vs. CON; F_OE_ = 71, vs. CON; F_OE_ = 7.607, vs. KD) Immunofluorescent (IF) staining for M1‐related biomarker (iba‐1 and iNOS) and M2‐related biomarkers (iba‐1 and Arg‐1) (B, E: F_OE_ = 6.250, vs. CON; F_OE_ = 6.875. vs. KD) in ROCK‐KD and ROCK‐OE BV‐2 cells. Proportion of CD86 (M1 cell) and CD206 (M2 cell) in BV‐2 cells (C, F: F_OE_ = 204.3, vs. CON; F_OE_ = 205.4, vs. KD; G: F_OE_ = 52.11, vs. CON; F_OE_ = 90.70. vs. KD). The expression of RhoA/ROCK signaling pathway in ROCK‐KD and ROCK‐OE BV‐2 cells. The expression of p‐MYPT1, p‐MLCK, RhoA (H) and ROCK1/2 (I) proteins expression; Statistics for expressions of p‐MYPT1 (J: F_OE_ = 62.30, vs. CON; F_OE_ = 53.18, vs. KD), p‐MLCK (K: F_OE_ = 114.7, vs. CON; F_OE_ = 48.30, vs. KD), RhoA (L: F_OE_ = 9.339, vs. CON; F_OE_ = 1.706, vs. KD), ROCK2 (M: F_OE_ = 13.48, vs. CON; F_KD_ = 16.96, vs. CON; F_OE_ = 1258, vs. KD) and ROCK1 (N) in different groups.

Lentiviral transfection was introduced to obtain the BV‐2 cell lines with ROCK‐KD and ROCK‐OE groups. The results of IF staining showed that co‐staining of iba‐1 and iNOS (biomarkers for M1) significantly increased in the ROCK‐OE cells, but decreased in the ROCK‐KD cells (Figure 6A,D). However, co‐staining of iba‐1 and Arg‐1 (biomarkers of M2) increased in the CON and ROCK‐KD groups, but decreased in the ROCK‐OE group (Figure 6B,E). The results of FCM showed that the proportion of CD86 significantly increased in the ROCK‐OE group in comparison with those in the CON and ROCK‐KD groups, while the proportion of CD206 significantly increased in the ROCK‐KD and CON groups in comparison with those in the ROCK‐OE group (Figure 6C,F,G).

The mRNA levels of inflammatory cytokines were also detected. The results showed that the proinflammatory cytokines (TNF‐α, CCL‐3, CCL‐5, iNOS) upregulated in the ROCK‐OE group than those in the CON and ROCK‐KD groups (Supporting Information: Figure 4A–D), while anti‐inflammatory cytokines (Fizz‐1, IL‐10, CD206, Arg‐1) upregulated in the ROCK‐KD and CON groups than those in the ROCK‐OE group (Supporting Information: Figure 4E–H).

We found that the expression levels of RhoA, p‐MLCK, p‐MYPT1, and ROCK2 proteins were markedly elevated in the ROCK‐OE cells in comparison with those in CON and ROCK‐KD groups. However, no statistical significance for the ROCK1 protein expression was observed among the different cell groups (Figure 6H–N).

## 4 DISCUSSION

In this study, we found that MEL reduced the frequency and severity of seizure in TLE modeling mice, indicating its neuroprotective role in epilepsy. Histological evidence suggested that MEL alleviated the apoptosis of hippocampus neuron in CA1 and CA3 regions. Interestingly, we found that after 7 days of MEL treatment, H&E and Nissl staining showed partial recovery of neurons in the treatment group, but there was no statistically significant difference, possibly because neurons had not yet fully recovered. TUNEL staining was statistically different, indicating that MEL reduced apoptosis within a short period of time. Furthermore, in vivo and in vitro studies proved that MEL regulated MG polarization, reducing the inflammatory responses by promoting the secretion of M2 anti‐inflammatory phenotypic markers and meanwhile inhibiting the secretion of M1 proinflammatory phenotypic markers.

MEL has been widely investigated in both clinical and fundamental sciences. The disorders in circadian rhythms significant increase the risk of neurological diseases.^37^, ^38^ Hablitz et al. found that MEL influenced circadian phase and electrical activity in the suprachiasmatic nucleus through G‐protein‐gated inwardly‐rectifying potassium (GIRK) channel activation, suggesting that the GIRK channel might be an important target for alternative therapies for circadian disorders.^39^ Another study demonstrated that the disruption of circadian innate immune homeostasis related to the abnormal rhythmic expression of Klf4 in aging macrophages.^40^

Some studies have proved the neuroprotective role of MEL in the neurological diseases, especially in neurodegenerative diseases, such as Alzheimer's Disease,^41^, ^42^ epilepsy,^43^, ^44^ and Parkinson's disease.^45^, ^46^, ^47^ This protection is closely related to the circadian properties of MEL.^48^ A clinical study found that MEL concentrations varied with circadian variation.^49^ Gubin et al. found that the application of MEL significantly improved glaucoma that was caused by circadian disruption.^50^ Molina‐Carballo et al. found that MEL level significantly increased in both febrile and epileptic children compared with that in healthy children, particularly when circadian rhythms alternated.^51^ Xi et al. found that MEL reduced trimethyltin chloride (TMT)‐induced neurotoxicity by inhibiting SERPINA3N‐mediated neuroinflammatory reaction and glial cell activation.^28^ These evidences suggest MEL may be served as an important mediator in CNS and a potential therapeutic target for diseases. However, although abundant evidences have proved that MEL could alleviate epileptic syndromes,^52^, ^53^ the clinical application of MEL on epilepsy treatment still has no substantial progress.

In clinical practice, TLE is one of the most common epilepsy phenotype in adults, but nearly half of patients with TLE are resistant to drug therapies.^54^, ^55^ Although the focal epilepsy usually originates from the regional brain, it is increasingly believed that TLE probably injuries the whole network of the brain.^56^ Therefore, the patients with TLE also experience a wide range of cognitive, mental, and behavioral disorders, interfering with quality of daily life.^57^ Currently, researches on epilepsy is progressing. For instances, the JNK signaling pathway was found to be involved in inflammatory responses to posttraumatic epilepsy.^58^ However, the detailed mechanisms are still needed to be investigate to provide useful information in therapies toward epilepsy. In this study, MEL significantly reduced the seizure frequency and severity in TLE modeling mice and improved cognition, learning and memory abilities. The results are similar with previously established investigations.

Mechanism studies about MEL have indicated that some signaling pathways have been involved in the pathogenesis of epilepsy. For instances, MEL was reported to play a neuroprotective role in KA‐induced hippocampal neuronal excitotoxicity by activating the neuronal PI3K/Akt pathway.^59^ The effect of MEL on the transformation of the polarization status of MG has been a hotspot in recent years. Small et al. found that IL‐6 activation had a significant impact on the inflammatory response of MG.^60^ However, the study about MEL in MG polarization is relatively rare in the area of epileptic diseases.

A study reported that MEL played an antioxidant and anti‐inflammatory role in neurodegeneration at the hippocampal site by altering the direction of microglial polarization.^61^ Additionally, it has been reported that MEL reduced the expression level of M1 markers and played a diverse role in the expression level of M2 phenotypic markers of early MG in the pathogenesis of depression.^62^ Besides, Zhou et al. found that MEL shifted M1 MG toward M2 through MT1/JAK2/STAT3/telomerase pathway and ameliorated periventricular white matter injury in rats.^63^ Li et al. discovered that MEL promoted MG polarization to M2 through STAT signaling pathway, therefore inhibiting neuroinflammation in parkinsonism‐related symptoms.^64^

In our study, we found that MEL influenced MG polarization probably through RhoA/ROCK signaling pathway. When neurons are injured, Rho is activated and induces the atrophy of growth cone and axon regeneration disorder. MEL is proved to promote axon growth by inhibiting Rho.^65^ RhoA can be activated after the LPS stimulation, and subsequently binds to ROCK. The complex increases the formation of calmodulin, upregulates the intracellular Ca^2^^+^ concentration, activates MLCK and therefore upregulates phospho‐MLCK (p‐MLCK) levels. At the same time, myosin light chain phosphatase (MLCP) inhibits p‐MLCK dephosphorylation, resulting in an increase of vascular endothelial permeability and barrier dysfunction.^66^, ^67^

We found that the expressions of RhoA, p‐MYPT1, and p‐MLCK decreased in KA + MEL and LPS + MEL groups in comparison with that in KA and LPS groups. It indicated that RhoA/ROCK signaling pathway involved in the MG polarization processes that might be induced by MEL. Xu et al. recently found that MEL had a therapeutic effect in advanced age‐related macular degeneration (AMD) by inhibiting macrophage polarization to M2 cells and simultaneously promoting it to M1 cells through RhoA/ROCK signaling pathway.^32^ However, there are still lack of evidences on the direct effect of MEL on ROCK1 and ROCK2. Our findings suggested that MEL had no effect on ROCK1 but inversely regulated the ROCK2 signaling pathway by downregulating the expression of the downstream molecules, p‐MYPT1, and p‐MLCK. In addition, we also found that the expressions of inflammatory marker for M1 phenotype decreased, while the expressions of marker for M2 phenotype increased in the KA + MEL group, indicating that MEL could alter the MG polarization in SE modeling mice. Meanwhile, in cytological experiments, ROCK‐OE reduced the protective effect of MEL, while the opposite results occurred in the ROCK‐KD cells. These results further demonstrated that MEL reduced SE mainly through ROCK2 signaling pathway.

## 5 CONCLUSION

In summary, we found that MEL played an antiepileptic role in the KA‐induced TLE modeling mice both in behavioral and histological levels. Meanwhile, MEL changed MG polarization to a M2 phenotype by inversely regulating the RhoA/ROCK2 signaling pathway. The results suggested that the MEL‐mediating regulation of RhoA/ROCK2 signaling pathway might be a potential important target in the treatment of epilepsy.

## AUTHOR CONTRIBUTIONS

**Pingping Li**: Conceptualization; formal analysis; investigation; methodology; writing—original draft. **Xuefei Ji**: Data curation; investigation; methodology; writing—original draft. **Ming Shan**: Investigation; resources. **Yi Wang**: Investigation; resources. **Xingliang Dai**: Resources; validation. **Mengyuan Yin**: Data curation; formal analysis; investigation. **Yunlong Liu**: investigation; resources. **Liao Guan**: Investigation; methodology. **Lei Ye**: Conceptualization; funding acquisition; supervision; wirtingreview and editing. **Hongwei Cheng**: Conceptualization; funding acquisition; project administration; supervision; writing—review and editing.

## CONFLICT OF INTEREST STATEMENT

The authors declare no conflict of interest.

## ETHICS STATEMENT

All animal handling and experimental protocols were reviewed and approved by the Institutional Animal Care and Use Committee and Ethics Committee of First Affiliated Hospital of Anhui Medical University (approval No: LLSC20200107).

## Supporting information

Supporting information.

Supporting information.

Supporting information.

Supporting information.

Supporting information.

Supporting information.

## Data Availability

All data relevant to the study are included in the article for figures and supplemental figures. Data are available from the corresponding author upon reasonable request.
