# Supplementary material for: Melatonin regulates microglial polarization to M2 cell via RhoA/ROCK signaling pathway in epilepsy
Source: Immun Inflamm Dis. 2023 Jun 14;11(6):e900. doi: 10.1002/iid3.900 (PMC10266134; doi:10.1002/iid3.900)
Supplement: Supplementary file 1 — Supporting information. [file IID3-11-e900-s005.docx]

**Supplementary Table 1.** Primer information for the qRT-PCR.

| Gene | Forward Primer | Reverse Primer |
| --- | --- | --- |
| iNOS | GAGACAGGGAAGTCTGAAGCAC | CCAGCAGTAGTTGCTCCTCTTC |
| TNF-α | GGTGCCTATGTCTCAGCCTCTT | GCCATAGAACTGATGAGAGGGAG |
| CCL-5 | CTG CTG CTT TGC CTA CCT CT | CGA GTG ACA AAC ACG ACT GC |
| CCL-3 | AAG GAT ACA AGC AGC AGC GAG TA | TGC AGA GTG TCA TGG TAC AGA GAA |
| Arg-1 | TGC AGA GTG TCA TGG TAC AGA GAA | TTG GCA GAT ATG CAG GGA GT |
| Fizz-1 | CCC TCC ACT GTA ACG AAG ACT C | CAC ACC CAG TAG CAG TCA TCC |
| IL-10 | GGC AGA GAA CCA TGG CCC AGA A | AAT CGA TGA CAG CGC CTC AGC C |
| Ym -1 | GAA CAC TGA GCT AAA AAC TCT CCT G | GAG ACC ATG GCA CTG AAC G |
| CD206 | GTG GTC CTC CTG ATT GTG ATA G | CAC TTG TTC CTG GAC TCA GAT TA |
| GAPDH | CATCACTGCCACCCAGAAGACTG | ATGCCAGTGAGCTTCCCGTTCAG |
