# Supplementary material for: Melatonin regulates microglial polarization to M2 cell via RhoA/ROCK signaling pathway in epilepsy
Source: Immun Inflamm Dis. 2023 Jun 14;11(6):e900. doi: 10.1002/iid3.900 (PMC10266134; doi:10.1002/iid3.900)
Supplement: Supplementary file 2 — Supporting information. [file IID3-11-e900-s001.docx]

**Supplementary Figure 2.** Core target sequences for shRNA and primers amplifying ROCK2**.**

shRNA Construct Sequences

| Gene | Sequence |
| --- | --- |
| ROCK2 | 1) 5’-GGTTTATGCTATGAAGCTT-3’ |
|  | 2) 5’-GCAGCAATTTCGATGACAT-3’ |
|  | 3) 5’-GCAACTGGCTCGTTCAATT-3’ |
|  | 4) 5’-GCACCTTGCAAAGTATATT-3’ |
| Control | 5’-TTCTCCGAACGTGTCACGT-3’ |

Primers for amplification

| Gene | Primer |
| --- | --- |
| ROCK2 | 1) 5’- CGCAAATGGGCGGTAGGCGTG -3’ |
|  | 2) 5’- CAGCGGGGCTGCTAAAGCGCATGC -3’ |
