# Supplementary figures and images for: Melatonin regulates microglial polarization to M2 cell via RhoA/ROCK signaling pathway in epilepsy
Source: Immun Inflamm Dis. 2023 Jun 14;11(6):e900. doi: 10.1002/iid3.900 (PMC10266134; doi:10.1002/iid3.900)

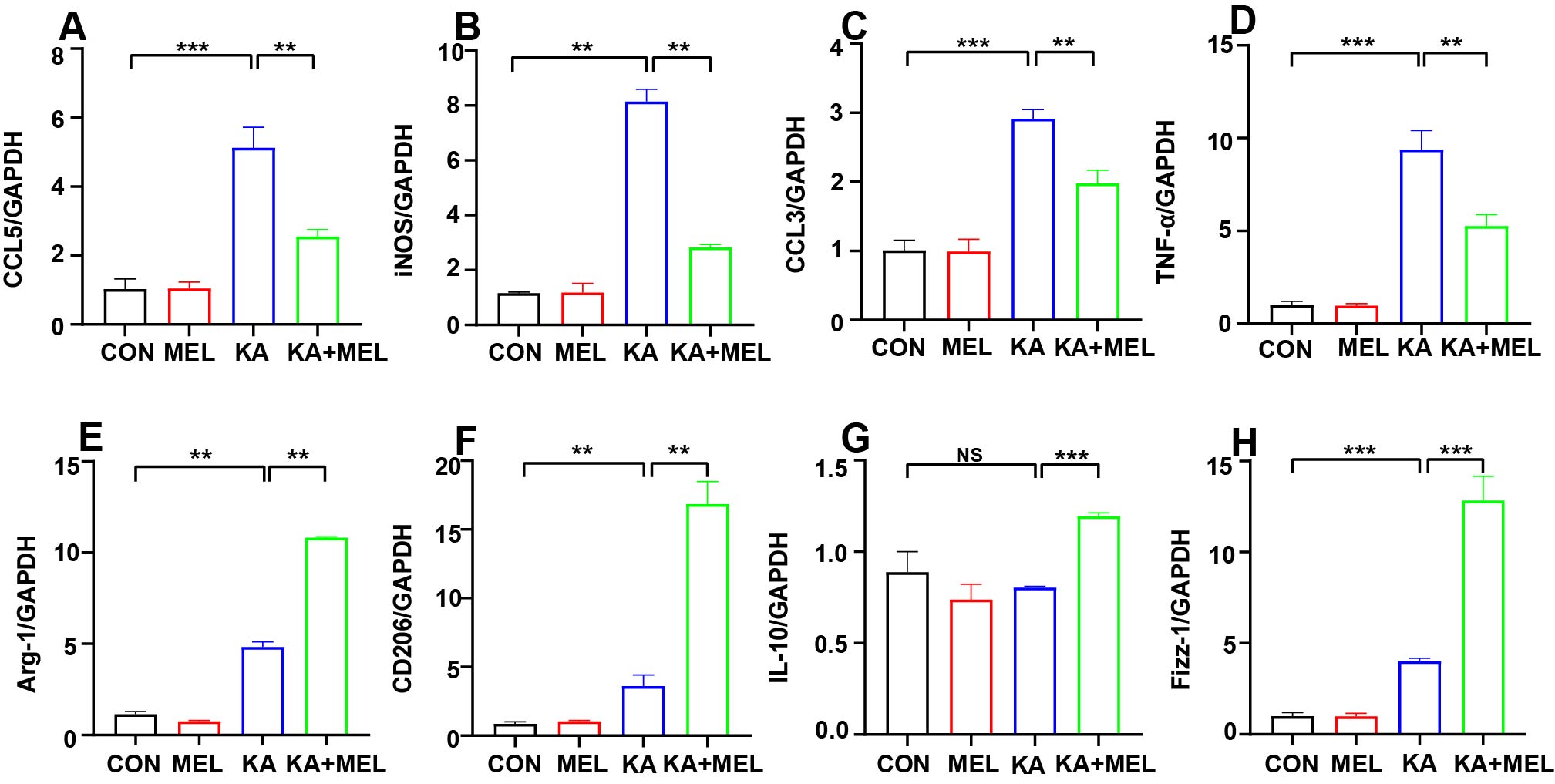

Supplement: Supplementary file 3 — Supporting information. [file IID3-11-e900-s003.jpg]

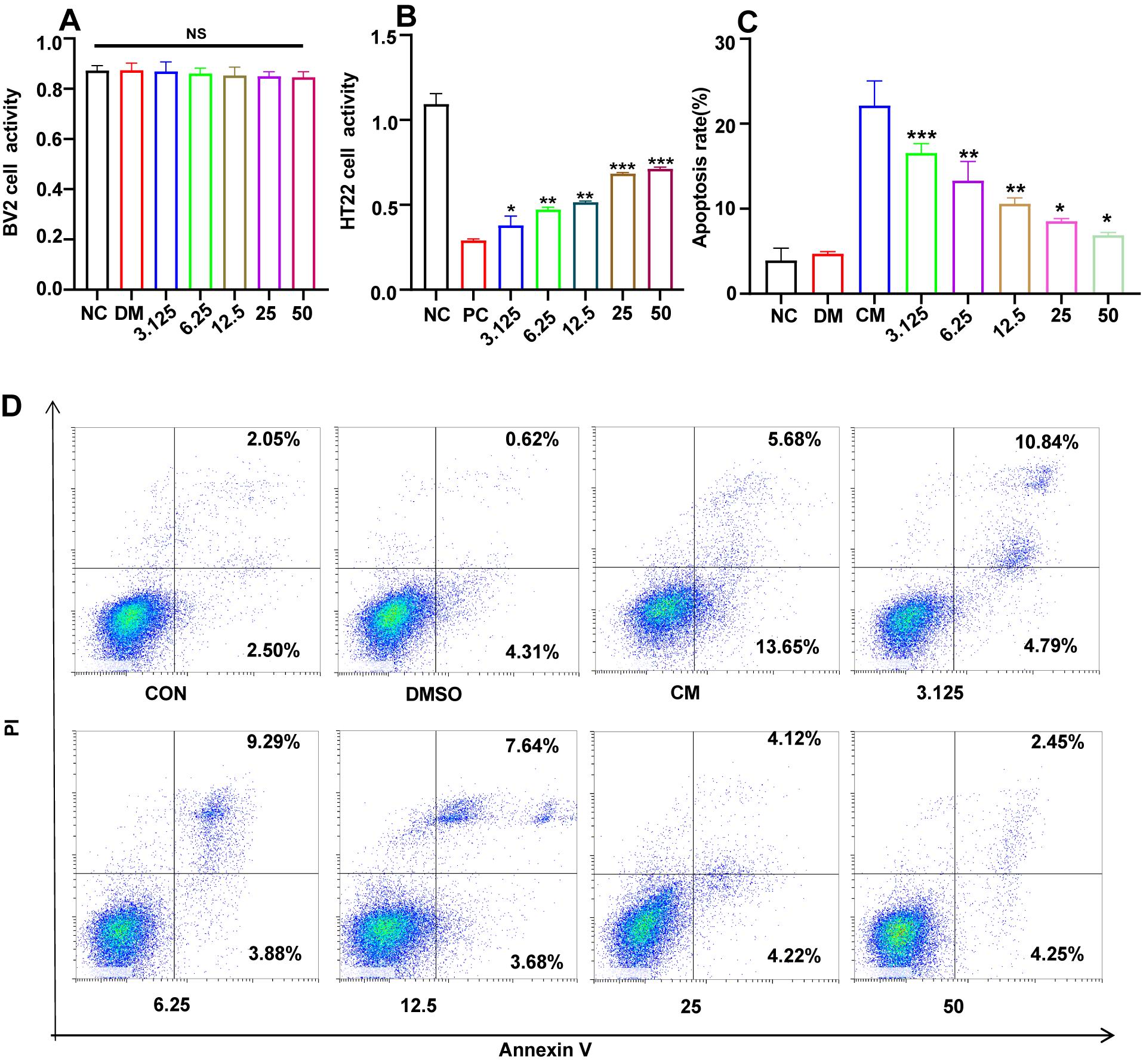

Supplement: Supplementary file 4 — Supporting information. [file IID3-11-e900-s004.jpg]

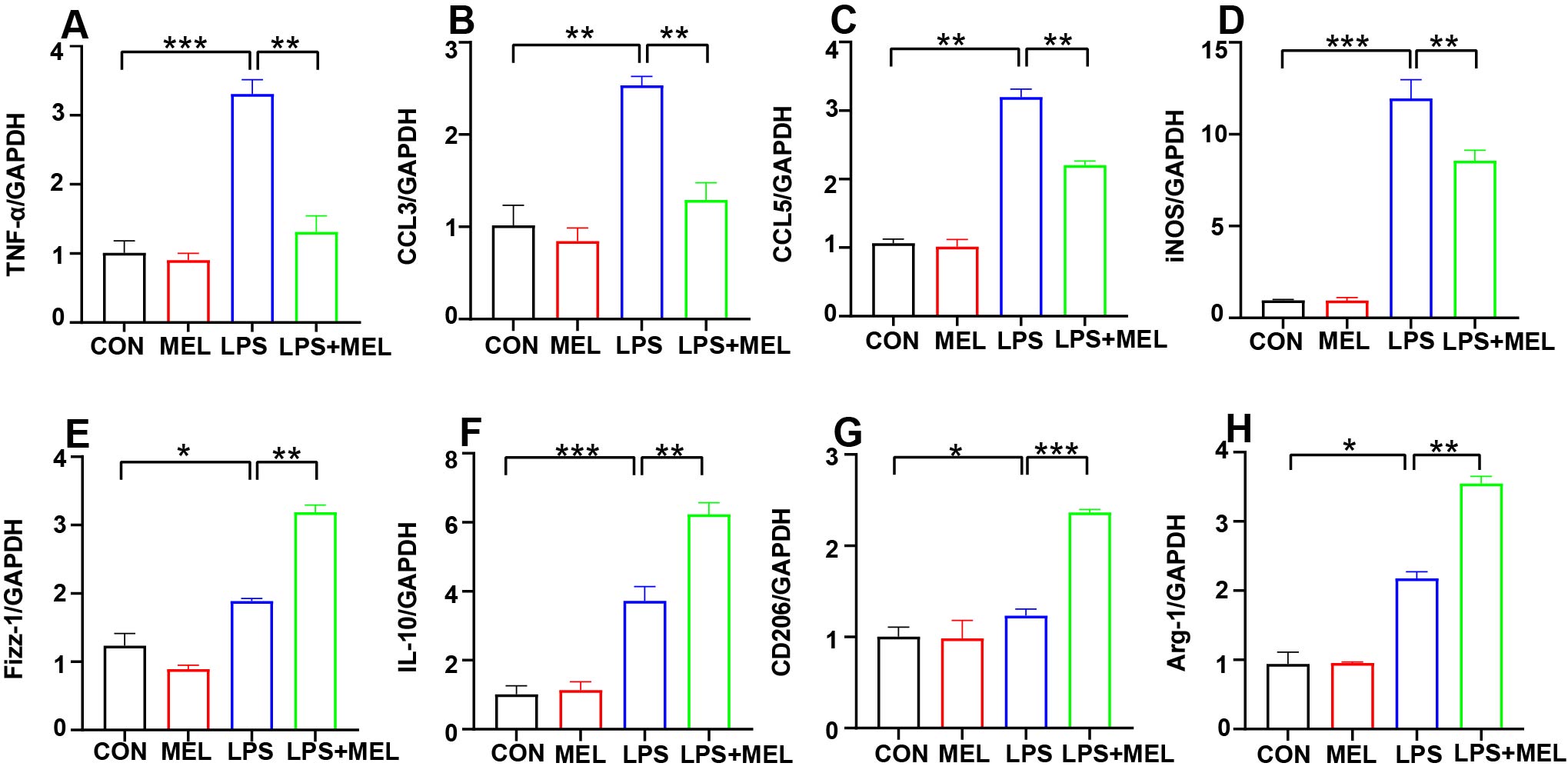

Supplement: Supplementary file 5 — Supporting information. [file IID3-11-e900-s006.jpg]

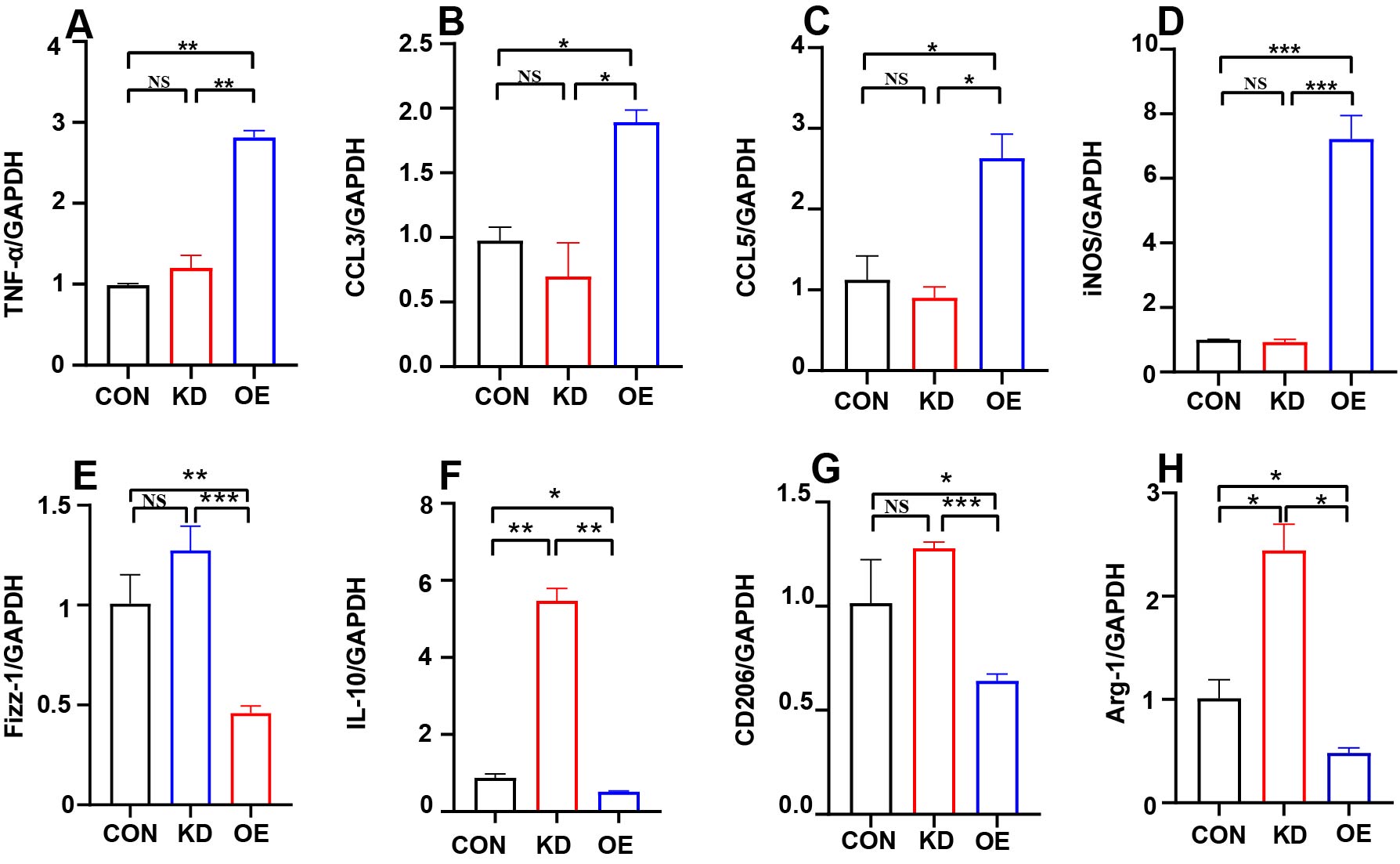

Supplement: Supplementary file 6 — Supporting information. [file IID3-11-e900-s002.jpg]
